# Influences of War on Medical Practice

**Published:** 1918-11-16

**Authors:** 


### The Hospital

The Workers' Newspaper of Administrative Medicine and Institutional Life, Administration, National Insurance and Health.

No. 1693, Vol. LXV. SATURDAY, NOVEMBER 16, 1918. PRICE TWOPENCE

### INFLUENCES OF WAR ON MEDICAL PRACTICE.

Medicine, like many other organisations and activities, has felt in many directions the impact and influence of the war, and it will in due time be an interesting study to follow the extent to which military experiences will prove helpful in civilian practice. The more appealing and dramatic of these experiences fall obviously into the department of surgery, but it may be doubted whether these will prove the most durable. Of course, surgical lessons of various orders have been taught by the war, and there is no need to minimise the value of them. But they relate to points of detail rather than to questions of principle, and their association is mainly with the differences, not with the similarities, which exist between military and civilian practice. Hence in the very nature of things such lessons must be largely transitory, and must disappear from the practical plane as the conditions which normally attend the surgical art are restored. The virulently infected wounds of the battlefield are a passing task, and they will not be repeated in civilian life. The various deformities which come within the sphere of orthopædic practice will doubtless make a longer claim, and the care of injured men and their restoration to a greater or less degree of functional efficiency will be a surgical obligation for many a day. Further, the methods which this obligation has promoted will certainly find an opportunity for application apart from wounded soldiers, as similar defects, though on a much narrower scale, present themselves in civilian practice. This department of work has been organised and developed with great thoroughness, and the experience and teaching which have resulted are likely to make an abiding mark both on professional opinion and professional work. There has been a decided advance in many directions, and this will have an enduring influence from which civilians as well as soldiers will reap the benefit.

On the more strictly medical side of practice special advantages will certainly follow the attention which has necessarily been directed to the mental and physical disturbances resulting from such influences as emotion and shock and nervous overstrain. First it has been demonstrated beyond question that a certain number of men possess a nervous system constitutionally incapable of sustaining the stresses of the battlefield. Though having the will to prove themselves good soldiers, they have not the power to adapt themselves to this new environment, and no training will fit them for the task. From such an experience there must necessarily arrive the conclusion that a parallel state of matters may exist in reference to claims arising in times of peace, and thus some patients who might well have been dismissed as wanting in earnestness and in resolution will be recognised as victims, in truth, of inability beyond the power of redemption. The obvious treatment in such conditions is not to urge to continued or repeated effort, but to provide a suitable environment. The "military misfit" will force the conviction of the existence of the "civilian misfit," and the advice found necessary for the one will repeat itself in the case of the other. Though both may in particular circles be classified as "weaker brethren," they have their own field of opportunity, and the task is to discover this suitable sphere.

More impressive and of much wider application is the experience that has been gained in reference to functional disorders of the nervous system, induced in particular by severe and sudden disturbances which, so far as can be judged, produce no appreciable anatomical change in the structures involved. "Shell shock" has taken its place as almost a classical term, and the opportunities of studying conditions so named have been numerous. These conditions have made a claim both on students of neurology and on students of psychology, and happily there has been a combined effort both to understand them and to treat them. Two broad conclusions have already established themselves. First it has been shown that the physical effects of the violence inflicted on the working of the nervous system may include manifestations which seem exactly to imitate gross organic injury. It has long been a medical commonplace that hysteria may simulate disease of almost any order, and such a doctrine finds its record in the usual text-books. But the war has made this teaching much more vital, and has extended the area of its application.

It has shown that even the elect may readily go astray in an attempt to distinguish between functional and organic nervous disease. Very naturally and properly the practitioner is warned against the danger of mistakenly regarding as "hysterical" symptoms which are really an expression of organic change, for such a blunder, other considerations apart, means a cruel injustice to the patient. Now, more than ever, it is clear that not less cruelty may follow from a mistake in the opposite direction. For, the paralysis and spasm due to functional disturbance will, if accepted by the practitioner as an announcement of structural mischief, be likely to continue and to become aggravated, with possibly permanent prejudice to the patient's capacity and usefulness. This consideration is the more urgent when a second lesson resulting from the experiences now under review is presented. This lesson, in brief, is that these physical consequences of nervous stresses can, if promptly recognised and suitably and energetically treated, be overcome without difficulty or delay. It is here that the psychological aspect of the condition becomes so important, for out of this has flowed the full application of methods that mean the restoration of lost functions in, it may be, minutes or hours rather than days. Again and again, and this by many observers, it has been shown that, given a correct diagnosis and suitable treatment, these disturbances can be banished almost immediately. The result has been the restoration either to the ranks or to civilian activities of numbers of men who might well have been condemned to a life of uselessness, and some of whom, as a matter of fact, had received this sentence. This teaching will certainly penetrate civilian practice, and will bring an atmosphere of confidence and certainty into a department of medicine where formerly there was a general feeling of helplessness and even of despair.

